# Undesirable dispersal via a river pathway of a single Argentine ant supercolony newly invading an inland urban area of Japan

**DOI:** 10.1038/s41598-023-47734-0

**Published:** 2023-11-30

**Authors:** Daisuke Hayasaka, Kenshin Kato, Masayoshi K. Hiraiwa, Hiro Kasai, Kazutaka Osaki, Retsushi Aoki, Takuo Sawahata

**Affiliations:** 1 Faculty of Agriculture, Kindai University, Nakamachi 3327-204, Nara, 631-8505 Japan; 2 Present Address: Fukui River and National Highway Office, Ministry of Land, Infrastructure, Transport and Tourism, Hanando-minami 2-14-7, Fukui, 918-8015 Japan; 3 Graduate School of Agriculture, Kindai University, Nakamachi 3327-204, Nara, 631-8505 Japan

**Keywords:** Ecology, Zoology, Ecology, Environmental sciences

## Abstract

Invasive ants pose a risk to human well-being and social/ecosystem stability. *Linepithema humile* Mayr is among the most damaging invasive ants worldwide. Most *L. humile* populations invade ports/wharfs isolated from surrounding landscapes, but unfortunately, a new population was discovered in an inland urban area (Nara Prefecture) of Japan in 2021. In this study, first, the supercolony type of the Nara *L. humile* population was identified via a hostility test, and then its distribution pattern was characterized. In aggression tests between *L. humile* from Nara and four supercolonies (haplotypes LH1, LH2, LH3, LH4), this ant showed extremely strong hostility against all supercolonies exept LH2, which was detected only in Japan in its introduced range. In Nara, *L. humile* was abundant in and around the urban river. Simulations revealed that using this environment for movement/dispersal increased the annual dispersal ability by 14 times compared with that achieved via ground (125 m), as mentioned in the literature. Therefore, river channels can serve as major pathways of long-distance dispersal for *L. humile* invading inland urban areas. Since applying chemical strategies around rivers is problematic, preventing *L. humile* from moving to rivers from initial invasion sites is crucial.

International trade and/or tourism are closely related to the shifts/movement of organisms (i.e., biological invasion)^1^. Among biological invaders, species that are introduced unintentionally pose a risk due to the obscurity of their invasion pathways and periods^2–4^. Therefore, the frequency of unintentional biological invasions has increased at an accelerated pace. Invasive species are recognized as one of the major obstacles to human well-being and the economy^5^. These species also have had an extreme impact on native ecosystems/communities within invaded areas^6,7^. Ants, including the yellow crazy ant (*Anoplolepis gracilipes* F. Smith)^8^ and the little fire ant (*Wasmannia auropunctata* Roger)^9^, are among the most invasive taxa^10–13^ due to their high fecundity and aggressiveness^14^. Notably, the Argentine ant (*Linepithema humile* Mayr), which is notorious as a nuisance^15^ and damaging invasive species^11^, has invaded every continent and many oceanic islands, excluding Antarctica, during the last 170 years^16,17^.

Along with several other invasive ant species, *L. humile* exhibits a special social structure, supercoloniality, in which many reproductive queens (polygynous) and numerous workers move freely among interconnected nests that are genetically similar due to common descent^11,18,19^. Additionally, this species has acquired a wide dietary breadth^20^. Therefore, *L. humile* has a competitive advantage over local communities of arthropods, including ants, within infested areas and subsequently causes species displacement^21–23^. Furthermore, ants can seriously influence agricultural and horticultural activities via “ant–aphid/scale insect mutualisms”^24–26^. Based on these findings, *L. humile* has been listed as one of the world’s 100 most damaging invasive species^27^ and invasive alien species (IAS) under the Invasive Alien Species Act of Japan^28^; thus, rapid and effective eradication of *L. humile* is desired^29,30^.

In Japan, four *L. humile* supercolonies (distinct in their haplotypes) have established colonies from east to west within only decades after the first detection in Hiroshima Prefecture in 1993^31–33^. This rapid establishment was due to unintentional introduction via the import of commodities. Most populations of *L. humile* first invade ports/wharfs that are isolated from surrounding landscapes but then secondarily translocate to a distant location, such as inland regions/areas, via main roadways and/or industrial roads with transported materials/goods^31,33–35^.

A new *L. humile* population was discovered in Nara City, Nara Prefecture (Japan), which is an inland regional city, in July 2021 (Fig. 1). A previous study indicated that *L. humile* with a small colony size and/or narrow distribution could easily be eradicated for up to 5 years after invasion, but its management would become extremely difficult 15 years after the initial discovery of the colony^36^. In fact, extirpation of *L. humile* populations introduced into Oi Wharf (Tokyo) in 2010 was achieved by 2015 through intensive chemical strategies (i.e., insecticidal baits and spraying)^23,29^. Thus, *L. humile* in Nara might also be relatively easily controlled if intensive strategies are implemented. However, *L. humile* from Nara has unfortunately been discovered in various landscapes, including not only urban zones but also watersides and paddy/dry fields. In particular, the ant is frequently observed along an urban river (Akishino River).

**Figure 1:**
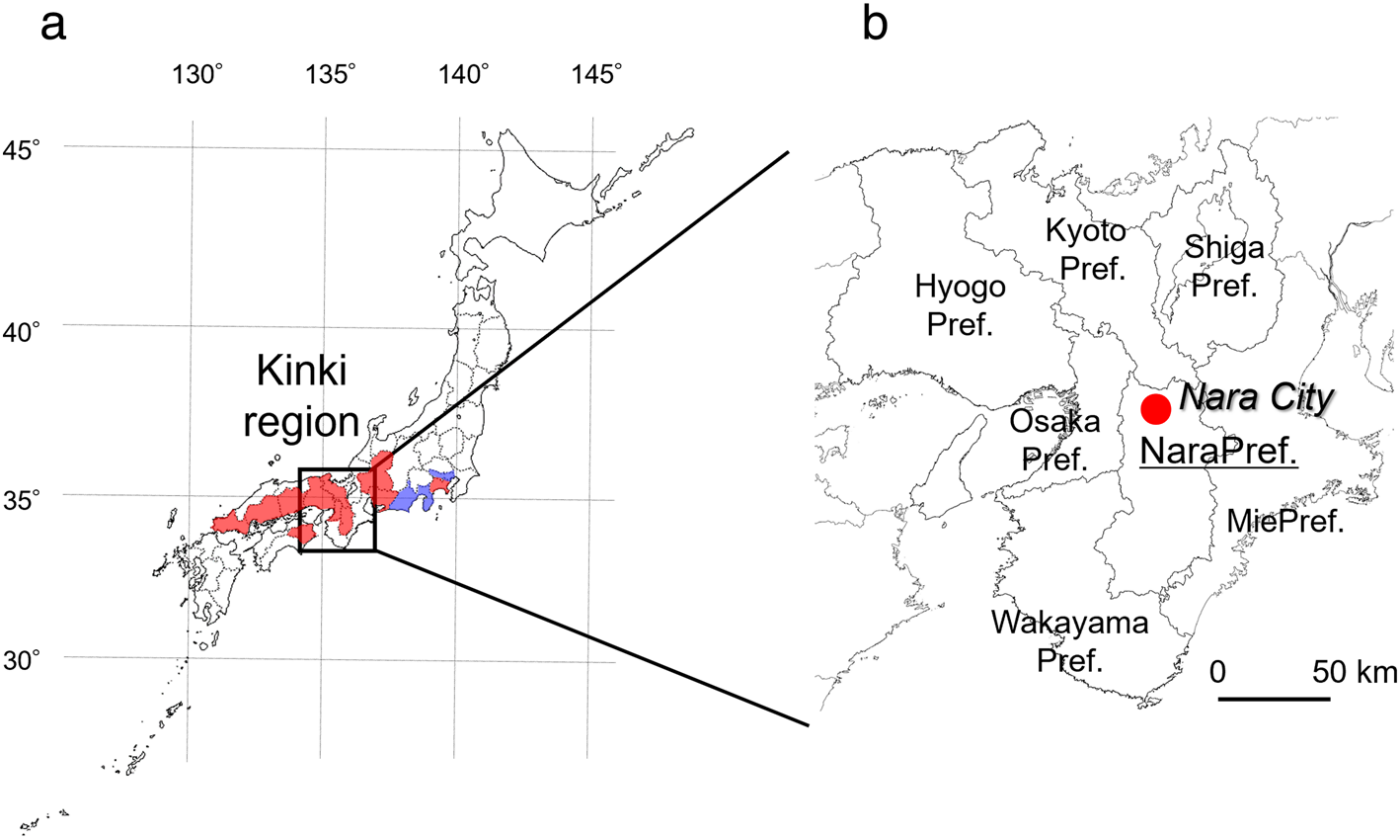
Location of the Argentine ant (*Linepithema humile*) newly invading Japan in 2021 (Nara City, Nara Prefecture). (**a**) In 2021, *L. humile* was established in 11 prefectures (red: Kanagawa, Aichi, Gifu, Nara, Kyoto, Osaka, Hyogo, Okayama, Hiroshima, Yamaguchi, and Tokushima)^31–33^ excluding two regions (blue: Tokyo and Shizuoka)^23,29^ where it had already been extirpated. (**b**) In the Kinki region, *L. humile* populations have already been detected in Hyogo (1999), Osaka (2007), and Kyoto (2009) prefectures.

There are two types of dispersal modes, anthropogenic transfer mediated by human activities (jump dispersal)^16,37^ and natural dispersal (inherent dispersal ability)^16,38–40^, for such supercolonial ants. Via the former mode, *L. humile* can spread annually on a local scale (several hundreds of metres to kilometres)^19^ to a transcontinental scale (several hundreds to several thousands of kilometres)^16,41^ through the transport of goods/materials. In contrast, the mode of natural dispersal can include either the diffusion of the colony on the ground via bud nests or the movement of small colony fragments rafting on rivers/watercourses. The annual inherent dispersal ability via the ground of *L. humile*, which does not exhibit nuptial flight behaviour^42^, ranges from 70 to 180 m (average of 125 m) in Japan^39^ and 150 m on average^16^ (minimum within ten metres^40^) in other regions/countries in the introduced range. On the other hand, the possibility that the annual dispersal distance of *L. humile* varies depending on the landscape structures within the invaded areas should not be overlooked. In the region of origin, *L. humile* frequently utilizes rivers for its natural dispersal^43^. In general, species that move/shift via rivers (i.e., lotic environments) have higher dispersal ability^44,45^ than those that move/shift via the ground, irrespective of taxonomic group. Therefore, rivers are a major pathway for the long-distance dispersal of species in the absence of competitors and can lead to distribution patterns similar to those expected under jump dispersal. Nevertheless, the detailed distribution and invasion route of *L. humile* from Nara remain unknown.

Herein, to clarify the distribution patterns of *L. humile* invading an inland urban city (Nara) corresponding to surrounding landscapes, we addressed the following two questions. 1. Does the *L. humile* population in Nara belong to one of the four supercolonies already described in Japan? 2. Has the Nara *L. humile* population expanded faster in the presence of water than via the ground? If so, how much faster?

## Results

### Hostile behaviours between intra- and inter-colony pairs of *L. humile*

**Figure 2:**
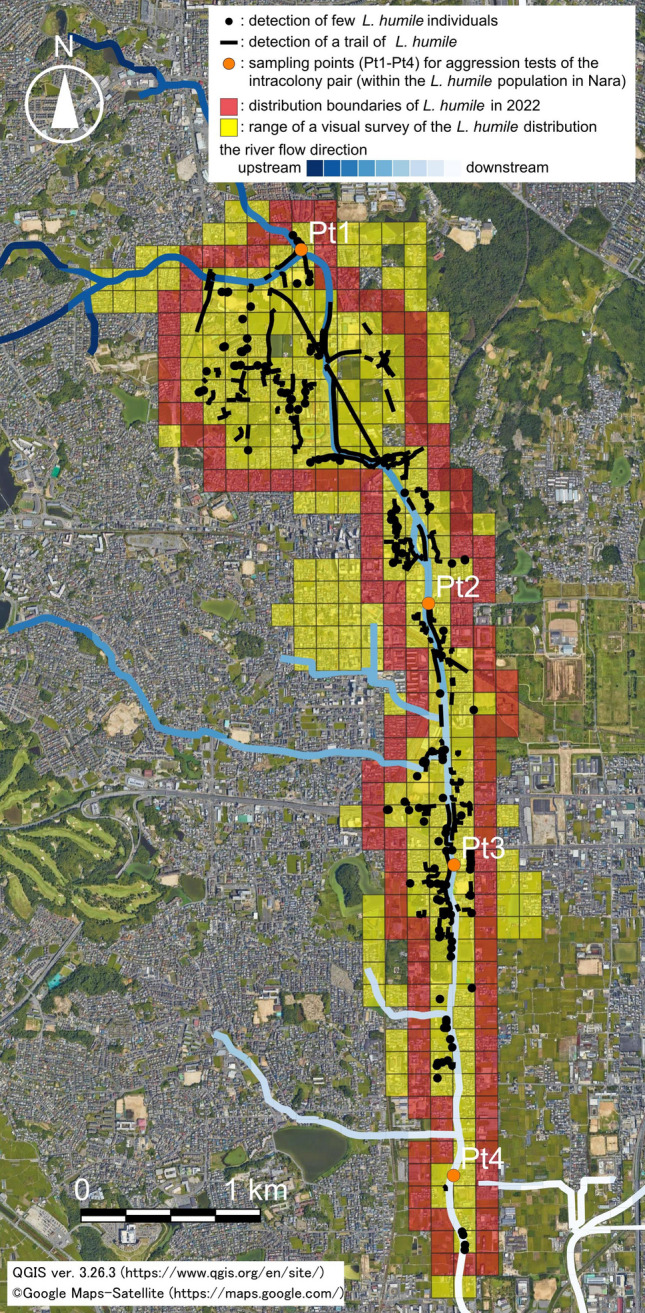
Distribution of invasive *L. humile* in an inland regional city (Nara City, Nara Prefecture) of Japan as of 2022 when field monitoring was conducted. The distribution map was created based on Google Maps using the free geographical software QGIS ver.3.26.3^48^. Considering the annual dispersal distance (70–180 m) of *L. humile* in Japan^39^, the occurrence ranges of ants in Nara and their distribution boundaries were divided into 125 m squares.

The distribution range of *L. humile* in Nara is shown in Fig. 2. In total, there were 132 cells (125 m squares/cells) in which *L. humile* was detected within the survey area. Thus, the aggression tests, which were performed not with individuals (‘one on one’) but with groups (‘five on five’) (cf. Roulston et al.^46^ and Sunamura et al.^47^), were conducted between *L. humile* individuals sampled from four points arbitrarily selected among the cells (intra-colony pair). These groups showed no hostile behaviours (aggression index = 0) towards each other throughout the trials (Fig. 2, Table 1). Thus, *L. humile* in Nara represents a single population. Given the result, workers from 87 of the total 132 cells, consisting of the cells at the centre of each line of *L. humile* and other observed cells, were selected to verify the presence/absence of hostility against the four *L. humile* supercolonies (haplotypes LH1-LH4) that genetically differed (inter-colony pair) through the same method used in the intra-colony pair tests (number of trials: 522 per pairing, 87 cells × 6 replicates, Supplementary Table S1). As a result, extremely strong hostility was found between *L. humile* from Nara and the LH1 (aggression index = 3.90 ± 0.316), LH3 (aggression index = 3.95 ± 0.234), and LH4 (aggression index = 3.94 ± 0.274) supercolonies. In fact, *L. humile* from Nara was aggressive towards these three supercolonies, even if alone, throughout the aggression tests. In contrast, the Nara *L. humile* population did not show hostile behaviours towards the LH2 supercolony in any trials (aggression index = 0; Table 2).

**Table 1:** Results of the ‘five-on-five’ aggression tests between the two colony pairs at the four sampling points (Pt) arbitrarily selected (intra-colony pair) among the cells of invasive *L. humile* detected in Nara, Japan (see subheading ‘Aggression tests’ for detailed methodology).

Intracolony pair	Interaction scores					Aggression index
	0	1	2	3	4	
Pt 1 vs. Pt 2	6	0	0	0	0	0
Pt 1 vs. Pt 3	6	0	0	0	0	0
Pt 1 vs. Pt 4	6	0	0	0	0	0
Pt 2 vs. Pt 3	6	0	0	0	0	0
Pt 2 vs. Pt 4	6	0	0	0	0	0
Pt 3 vs. Pt 4	6	0	0	0	0	0

Aggression indices (mean) were calculated based on the interaction scores in each colony pair consisting of six replicates each.

**Table 2:** Results of the ‘five-on-five’ aggression tests between the *L. humile* population in Nara and the four supercolonies (LH1, LH2, LH3, LH4) with different haplotypes (inter-colony pair) sampled from Kobe City, Hyogo Prefecture, using the same method for the intra-colony pairs shown in Table 1. The number of trials was 522 per pairing (87/132 cells × 6 replicates).

Intercolony pair	Interaction scores					Aggression index
	0	1	2	3	4	
vs. LH1	0	0	3	45	474	3.90 ± 0.316
vs. LH2	522	0	0	0	0	0
vs. LH3	0	0	1	26	495	3.95 ± 0.234
vs. LH4	0	0	5	21	496	3.94 ± 0.274

Aggression indices (mean ± SD) were calculated based on the interaction scores in each colony pair consisting of six replicates each.

### Distribution patterns of the *L. humile* population invading Nara

**Figure 3:**
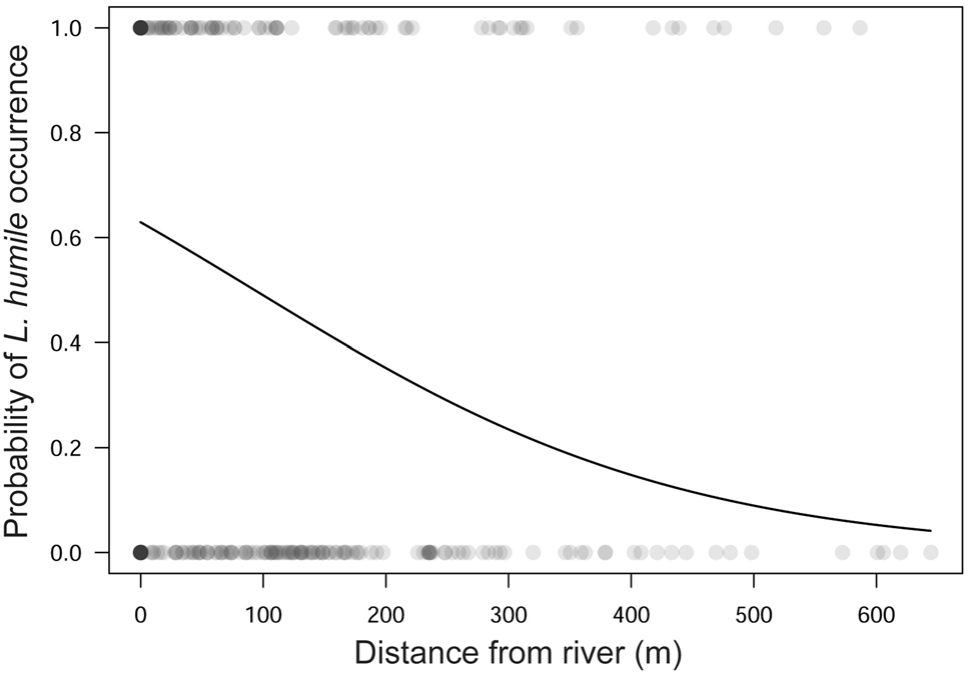
Relationship between the occurrence of invasive *L. humile* in Nara (an inland regional city) and the distance from the river. The line indicates the regression line estimated by the GLM.

The *L. humile* population in Nara was not concentrically distributed but spread vertically along the urban river (Akishino River). The closer to the river, the more abundant *L. humile* was (Fig. 3; distance from the river, coefficient = −0.0043, *z* = −4.096, *P* < 0.001; spatial autocorrelation, coefficient = −0.0004, *z* = 8.805, *P* < 0.001, GLM). The distribution distance of *L. humile* along the river and the linear distance from the northern to southern ends of the distribution were approximately 6100 and 5740 m, respectively. On the other hand, the direct distance from the riverbanks to the inner occurrence points of *L. humile* ranged from 10 to 875 m. The ant was also detected along herbaceous avenues, around street trees, and in agricultural lands near the river. In the upstream section of the river, *L. humile* was widely spread from the river to inner areas, but it was limited to near the river downstream despite the landscapes being similar to those upstream.

**Figure 4:**
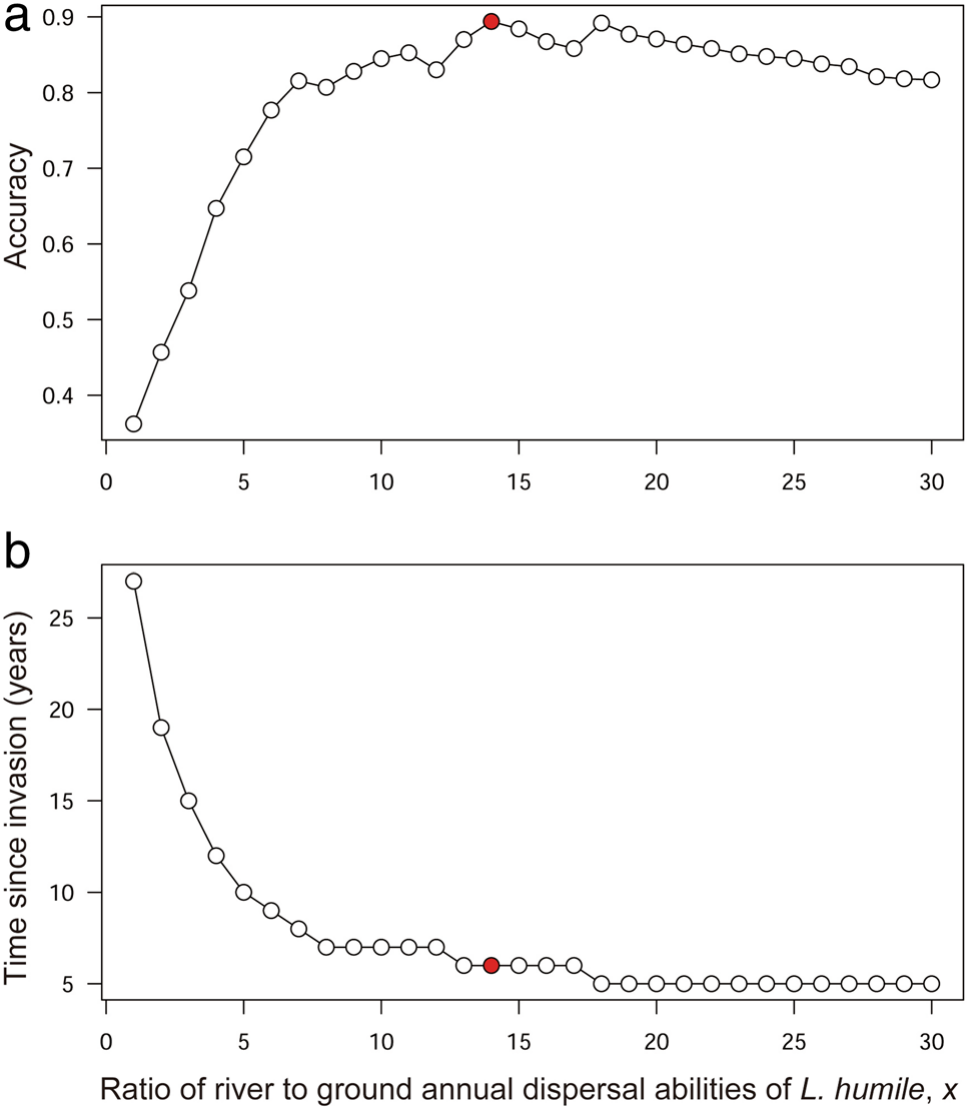
Relationships between the ratio of river to ground annual dispersal abilities of *L. humile*, *x*, and the highest accuracy (**a**) and time since invasion (**b**) in the cellular automaton simulation for each *x* value. The red points indicate the simulation result with the highest accuracy among all simulations.

We conducted 3960 simulations for the distribution expansion of *L. humile*, and the highest 10 accuracies were between 0.86 and 0.89. In the top 10 simulations with the highest accuracies, the ratio of river to ground for dispersal ability, *x*, ranged from 13 to 20. The estimated invasion periods from these simulations ranged from six to eight years ago. On the other hand, the highest accuracy was 0.36, assuming that the annual dispersal ability of *L. humile* was the same between the ground and river pathways (Fig. 4a, the ratio of river to ground for dispersal ability, *x* = 1; dispersal ability via the river 125 m/year). The invasion period of *L. humile* in Nara based on this simulation was estimated to have begun approximately 28 years ago (Fig. 4b).

**Figure 5:**
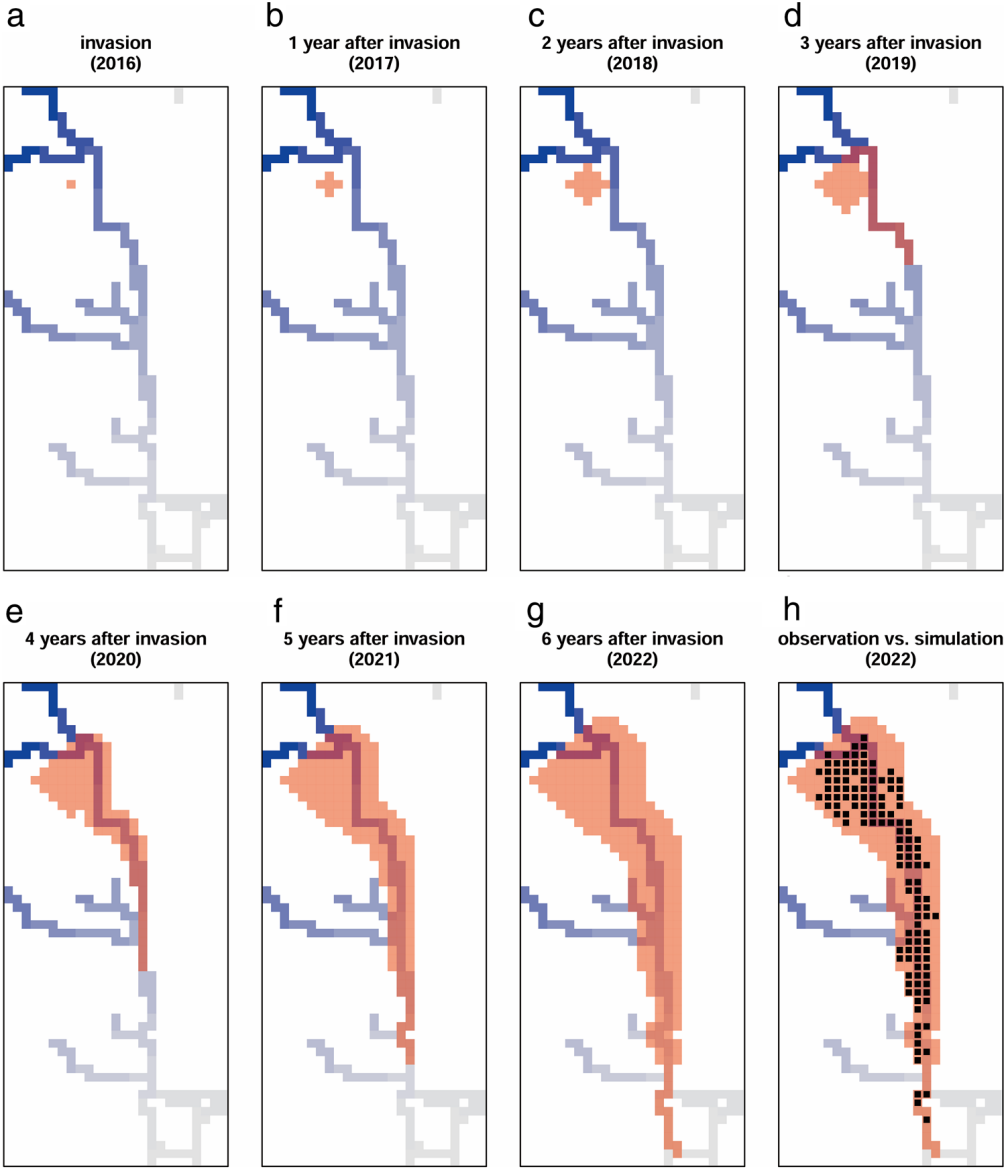
The simulation results with the highest accuracy (the ratio of river to ground annual dispersal abilities of *L. humile*, *x* = 14; dispersal ability via the river: 1750 m/year). The red area indicates the simulated distribution area of *L. humile*. Black cells indicate the actual observed distribution area of *L. humile*. The blue (upstream) to grey (downstream) gradient indicates the direction of river flow.

The highest accuracy simulation (accuracy = 0.89) was obtained when assuming that the annual dispersal ability via the river was 14 times greater than that via the ground (Figs. 4a and 5h, the ratio of river to ground for dispersal ability, *x* = 14; dispersal ability via the river 1750 m/year). This simulation showed the possibility that *L. humile* first invaded the upstream area of the river and then dispersed via the ground for the first two years (Fig. 5a–c). After three years, *L. humile* reached the river, and then, its distribution range rapidly expanded (Fig. 5d), reaching the current distribution shape up to six years after the initial invasion (Fig. 5e–h).

## Discussion

Understanding the invasion/distribution patterns of organisms and their ecological characteristics/identity is fundamental to their effective control/extermination. Among invasive species such as *L. humile* with different ecological properties for each supercolony (e.g., insecticidal sensitivity^49^, dietary breadth^20^), conducting preliminary hostility tests and genetic analysis is particularly desirable. Our research revealed the undesirable dispersal via rivers of a single *L. humile* population invading an inland urban area.

There is no doubt that the invasion success of *L. humile* is predominantly attributable to its supercoloniality and high aggressiveness^50–53^. *Linepithema humile* forms a supercolony among populations with the same haplotype but exhibits hostile behaviour towards populations with different haplotypes^11,18,19^. Furthermore, populations are aggressive despite sharing the same haplotype when this sharing is due to chance rather than a common origin^41^. Aggression tests of the intra-colony pairs of *L. humile* revealed that a single supercolony of the ant has widely invaded Nara (Table 1). In addition, the possibility that *L. humile* in Nara is the same as the LH2 supercolony (i.e., nestmates) with low insecticide fipronil sensitivity^49^ was shown via inter-colony pair tests (Table 2). Notably, the LH2 supercolony of *L. humile* has been detected in Hyogo, Tokushima, and Shizuoka prefectures but not outside Japan^31^. Therefore, the *L. humile* population introduced into Nara might have originated in these prefectures.

Two additional factors determining the successful invasion of this species, especially its distribution expansion, are landscape structure and social infrastructure^54^. In Nara, *L. humile* is frequently found within and around the river (Figs. 2, 3). Similar findings were reported in other regional cities of Japan (e.g., Iwakuni City, Yamaguchi Prefecture)^39^. Unsurprisingly, *L. humile* tends to prefer lowland waterside environments, including rivers, for population expansions regardless of the native/introduced ranges^55,56^ because this species predominantly inhabits flat, expansive floodplains in its region of origin^43^. Regarding its high-frequency occurrence around lotic environments, *L. humile* also often inhabits sites with high humidity near rivers as a response and adaptation to dry and/or hot environments^56,57^, including urban and/or disturbed areas. Furthermore, the successful invasion and inhabitation of biological invaders such as *L. humile* depend on the presence/absence or strength of negative interspecific interactions such as competition^58,59^ in invaded areas. The possibility that river channels function as effective corridors for *L. humile* invasion and subsequent expansion should not be overlooked because of the lack of competitor species against *L. humile* in Japan^60^. Our findings strongly highlight the importance of water landscape structures as its long-distance dispersal pathways.

The inherent dispersal ability of *L. humile* with no nuptial flights^42^ (natural dispersal via the ground by bud nests and/or via river rafting/floating) is approximately three orders of magnitude lower than its jump dispersal associated with transportation vehicles^16,39,61^. The time since invasion via the river of *L. humile* detected in Nara in 2021 was approximately six years in the simulation estimation with the highest accuracy (0.89) (Figs. 4, 5). Additionally, its predicted distribution areas were very similar to the real ones (Fig. 5h). On the other hand, despite the first detection of *L. humile* in Japan (Hiroshima Prefecture) in 1993, its local invasion of Nara was estimated to have already started 28 years ago when assuming no distribution expansion of *L. humile* via the river (simulation accuracy: 0.36), which is not realistic. Therefore, undesirable long-distance dispersal would have occurred within a shorter period, as expected under jump dispersal via the import/transport of goods/materials, than with expansion via the ground when *L. humile* invaded the lotic environment/landscape. In addition, the population dynamics of *L. humile* after invasion may depend on the vegetation structure within the infested areas because of ant–aphid/scale insect mutualisms^24–26^. *Linepithema humile* in Nara will widely invade from the river to various landscapes in inner urban areas sooner or later due to landscape structures similar to those upstream even though downstream of the river, its occurrence is currently limited to the area surrounding the river channel (Fig. 2). In our study, the annual dispersal ability of *L. humile* via the river was 14 times (1750 m) greater than the inherent dispersal distance of *L. humile* via the ground (125 m)^39^. However, it should be noted that the annual dispersal distance of *L. humile* used in this study was based on that of an *L. humile* supercolony (LH1)^31^, which is genetically different from the ant population in Nara. The possibility that the inherent dispersal ability of *L. humile* differs considerably not only among sites and/or years but also among supercolonies cannot be ruled out^14,19^. Therefore, continuous and detailed (e.g., records of the occurrence of *L. humile* by smaller square cells) monitoring of the distribution tendencies of *L. humile* in Nara is essential for estimating its rate of dispersion with more precision.

In conclusion, preventing *L. humile* from moving to rivers from the initial invasion sites when introduced into sites near rivers is essential because chemical strategies around rivers are problematic (e.g., undesirable exposure of aquatic animals). Additionally, to restrict the establishment and subsequent dispersal of *L. humile* after invasion in lotic environments, we suggest that it would be effective to continuously reduce the density of its mutualistic partners (i.e., honeydew-producing insects) via vegetation management, despite this not being considered in detail in this study. Nevertheless, as mentioned before, *L. humile* eradication is generally achievable up to 5 years after invasion^36^, but this may be difficult if the ant invades river habitats.

## Materials and methods

### Distribution survey of the *L. humile* population invading Nara

This study was conducted in central Nara City, Nara Prefecture, Japan (34° 41′ 38.0″ N, 135° 46′ 57.0″ E) (Fig. 1), from July to December 2022. To understand the present distribution range of *L. humile* in Nara and estimate its invasion period, the occurrence of this species was monitored widely, except in restricted zones such as natural monuments, world heritage sites, shrines, temples, factory premises, and railway tracks, by a visual survey. Here, given that the annual on-ground dispersal distance of *L. humile* in Japan is estimated to be 125 m^39^, the occurrence range of *L. humile* in Nara was divided into 125 m squares (cells). The presence/absence of the ant in one or more outwards cells was also monitored if *L. humile* was present, and then its distribution boundaries were determined. Although a visual survey was implemented once per cell throughout the study period, cells without the detection of *L. humile* during the first monitoring period were surveyed again after a month. Location coordinates at which *L. humile* workers were present irrespective of the number of ant individuals (i.e., few or a line) were recorded using a GPS receiver (Garmin Oregon 300, Garmin Ltd., Kansas, USA). Then, a distribution map of the Nara *L. humile* population was generated in Google Maps using GIS software QGIS ver.3.26.3^48^.

### Sampling of *L. humile* workers

Multiple *L. humile* workers were collected using a fluke tube from arbitrary points within the cells in which the ant was present for the hostility test. The hostility tests were conducted to determine whether *L. humile* individuals from different nests belonged to the same supercolony with shared genetic structure (the same haplotype) (see subheading ‘Aggression tests’ for the method of hostility tests against *L. humile*). Workers were collected from only the cell at the centre of the line when a line of this species was found across multiple cells since there was not a line of *L. humile* that showed hostility among the cells. In addition, because all *L. humile* supercolonies in Japan (haplotypes LH1, LH2, LH3, and LH4) were detected at Kobe Port, Hyogo Prefecture, individuals from the four colonies were collected from multiple points (see Seko et al.^19^ for the distribution ranges of each supercolony). Then, aggression tests of intra-colony (within *L. humile* from Nara) and inter-colony (*L. humile* in Nara versus the four supercolonies) pairs were performed.

### Aggression tests

From September to December 2022, to determine the supercolony type to which *L. humile* in Nara belonged, a hostility test that allowed the detection of antagonistic supercolonies (‘five-on-five’ aggression test) was performed largely following Sunamura et al.^47^ and Roulston et al.^46^ with slight modification. The test was conducted in our laboratory and/or the field. Five workers of *L. humile* from the two colonies (intra-colony: within the *L. humile* population in Nara; inter-colony: Nara population versus four supercolonies with different haplotypes in Kobe) were introduced into a Petri dish (5.2 cm in diameter) and then separated by overlaying a top cover with a partition in the centre. Workers in each colony were held for 5 min for acclimation. Afterwards, the partition was removed, and then a hostility test was started. In addition, the side of each Petri dish was coated with talcum powder to prevent the escape of tested individuals. Interactions among introduced workers (i.e., presence/absence of a hostile behaviour) were observed for 10 min, and a behavioural (interaction) score with 5 levels was assigned (0: ignore, 1: touch (prolonged antennation), 2: avoid, 3: aggression (lunging, pulling, or biting), and 4: fight (prolonged aggression)). Hostility between the two colonies was defined as a score of 3 or 4. Six replicates of the aggression tests for all intra- (within the *L. humile* population in Nara) and inter-colony (Nara *L. humile* population versus four supercolonies) pairs were performed. The highest interaction score for each trial observed between the colony pairs during a 5–10 s scan every 2 min after the start of the experiment was recorded from six dishes (replicates). The highest score for each trial observed in 10 min was averaged across trials within each colony pair, and this average was used as the aggression index for the colony pair. This means that the higher the aggression index was, the more the personality differed between the colony pairs of *L. humile* (i.e., populations) and the lower the index; both pairs could be similar and/or the same (i.e., nestmates).

### Statistical analyses for the dispersal patterns of *L. humile* in Nara

To test whether *L. humile* in Nara was more abundant closer to the rivers, we fit a generalized linear model (GLM with binomial error and a logit link), where the presence/absence of the ant was the response variable. The explanatory variables were distance from the river and spatial autocorrelation. Because the cells within the study area were not independent, the effects of spatial autocorrelation were considered^62^.

To verify whether dispersal via rivers could explain the present distribution range of *L. humile* in Nara, we used a simple cellular automaton model^63^. A digital space consisting of 125 m square cells including the study area was constructed (57 × 25 = 1425 cells). Each cell was classified according to the presence or absence of rivers based on the data in Fig. 2. First, *L. humile* invades any one cell (step 0; 0 years after invasion). *Linepithema humile* within its distribution cell disperses deterministically via the ground and/or river in a year (1 step). Dispersal via the ground is performed in all cells in which *L. humile* is present, and the ant expands its distribution by one cell from the current distribution cell to the top, bottom, left and right after one year. The number of cells into which *L. humile* disperses via the river is determined by the ratio of river to ground dispersal abilities, *x*. This dispersion occurs when the cell into which the ant disperses via the ground is a cell containing a river. In this case, *L. humile* extends its distribution by an additional *x*-1 cells from upstream to downstream (total distribution expansion *x* cells/year). These steps were repeated until all the sites where *L. humile* was observed in fields were within the simulated distribution area. The accuracy of the final simulated distribution map was evaluated by comparing it to the actual distribution map. The accuracy index was calculated as follows:

$$Accuracy=\frac{Number\, of\, correctly\, predicted\, cells}{Total\, number\, of\, cells}.$$

The accuracy was one when all cells were correct, whereas it was zero when all cells were incorrect. Simulations were conducted by varying *x* from 1 to 30 in increments of one. For each value of *x*, we selected the initial distribution position from one of the cells where *L. humile* was observed in the field (132 cells). The accuracy was then calculated after performing simulations. The total number of simulations was 3960 (30 × 132), and then the accuracy of each simulation was assessed.

The GLM and cellular automaton model were fit and simulated using R software version 4.2.1^64^. Spatial autocorrelation was calculated by using the “spdep” package^65^.

### Supplementary Information

Supplementary Information 1.

Supplementary Information 2.

Supplementary Information 3.

Supplementary Information 4.

## Data Availability

Raw data for this study (i.e., maximum hostility score in the aggression tests between the *L. humile* population in Nara and the four supercolonies with different genotypes sampled from Kobe City, Hyogo Prefecture) and the R script for the cellular automaton model are included in the main manuscript and supplementary files.
