# Supplementary material for: Undesirable dispersal via a river pathway of a single Argentine ant supercolony newly invading an inland urban area of Japan
Source: Sci Rep. 2023 Nov 30;13:21119. doi: 10.1038/s41598-023-47734-0 (PMC10689755; doi:10.1038/s41598-023-47734-0)
Supplement: Supplementary file 4 — Supplementary Information 4. [file 41598_2023_47734_MOESM4_ESM.docx]

Undesirable dispersal via a river pathway of a single Argentine ant supercolony newly invading an inland urban area of Japan

Daisuke Hayasaka, Kenshin Kato, Masayoshi K. Hiraiwa, Hiro Kasai, Kazutaka Osaki, Retsushi Aoki and Takuo Sawahata

**Supplementary table S1**. Raw data for the maximum hostility score through the aggression tests between *Linepithema humile* (*Lh*) population in Nara and the four supercolony (LH1, LH2, LH3, and LH4) with different genotypes collected in Kobe, Hyogo Prefecture, Japan.

| *Lh*_Nara_ID | versus supercolony  (LHx) | Maximum hostility score between *Lh* in Nara and the four supercolony (LHx) in each trial | | | | | |
| --- | --- | --- | --- | --- | --- | --- | --- |
|  |  | 1st | 2nd | 3rd | 4th | 5th | 6th |
| 1 | LH1 | 4 | 4 | 4 | 4 | 3 | 4 |
| 2 | LH1 | 4 | 4 | 4 | 4 | 4 | 4 |
| 3 | LH1 | 4 | 4 | 4 | 4 | 4 | 4 |
| 4 | LH1 | 4 | 4 | 4 | 4 | 4 | 4 |
| 5 | LH1 | 4 | 4 | 4 | 4 | 4 | 4 |
| 6 | LH1 | 4 | 3 | 3 | 4 | 4 | 4 |
| 7 | LH1 | 4 | 4 | 4 | 4 | 4 | 4 |
| 8 | LH1 | 3 | 3 | 3 | 4 | 4 | 4 |
| 9 | LH1 | 4 | 4 | 4 | 4 | 4 | 3 |
| 10 | LH1 | 4 | 4 | 4 | 2 | 4 | 4 |
| 11 | LH1 | 4 | 4 | 4 | 4 | 4 | 4 |
| 12 | LH1 | 4 | 4 | 4 | 4 | 4 | 4 |
| 13 | LH1 | 3 | 3 | 4 | 4 | 4 | 4 |
| 14 | LH1 | 4 | 4 | 4 | 4 | 4 | 4 |
| 15 | LH1 | 4 | 3 | 4 | 4 | 4 | 4 |
| 16 | LH1 | 4 | 4 | 4 | 4 | 4 | 4 |
| 17 | LH1 | 4 | 4 | 4 | 4 | 4 | 4 |
| 18 | LH1 | 4 | 4 | 4 | 4 | 4 | 4 |
| 19 | LH1 | 4 | 4 | 4 | 4 | 4 | 4 |
| 20 | LH1 | 4 | 3 | 4 | 4 | 4 | 4 |
| 21 | LH1 | 4 | 4 | 4 | 4 | 4 | 4 |
| 22 | LH1 | 4 | 4 | 4 | 4 | 4 | 4 |
| 23 | LH1 | 4 | 4 | 4 | 4 | 4 | 4 |
| 24 | LH1 | 4 | 4 | 3 | 3 | 4 | 3 |
| 25 | LH1 | 4 | 4 | 4 | 4 | 4 | 4 |
| 26 | LH1 | 4 | 4 | 3 | 4 | 3 | 4 |
| 27 | LH1 | 4 | 4 | 4 | 4 | 4 | 4 |
| 28 | LH1 | 4 | 4 | 4 | 4 | 4 | 4 |
| 29 | LH1 | 4 | 4 | 4 | 4 | 4 | 4 |
| 30 | LH1 | 4 | 4 | 4 | 4 | 4 | 4 |
| 31 | LH1 | 4 | 4 | 4 | 4 | 4 | 4 |
| 32 | LH1 | 4 | 4 | 4 | 4 | 4 | 3 |
| 33 | LH1 | 4 | 4 | 4 | 4 | 4 | 4 |
| 34 | LH1 | 4 | 3 | 4 | 4 | 4 | 4 |
| 35 | LH1 | 4 | 3 | 4 | 4 | 3 | 4 |
| 36 | LH1 | 4 | 3 | 4 | 3 | 4 | 4 |
| 37 | LH1 | 4 | 4 | 4 | 4 | 4 | 4 |
| 38 | LH1 | 4 | 4 | 4 | 4 | 4 | 4 |
| 39 | LH1 | 4 | 4 | 4 | 4 | 4 | 4 |
| 40 | LH1 | 4 | 4 | 4 | 4 | 4 | 4 |
| 41 | LH1 | 4 | 4 | 4 | 4 | 4 | 4 |
| 42 | LH1 | 4 | 4 | 4 | 3 | 4 | 4 |
| 43 | LH1 | 4 | 4 | 4 | 4 | 4 | 4 |
| 44 | LH1 | 4 | 4 | 4 | 4 | 4 | 4 |
| 45 | LH1 | 4 | 4 | 4 | 4 | 4 | 4 |
| 46 | LH1 | 4 | 4 | 4 | 4 | 4 | 4 |
| 47 | LH1 | 4 | 4 | 4 | 4 | 4 | 4 |
| 48 | LH1 | 4 | 4 | 4 | 4 | 4 | 4 |
| 49 | LH1 | 4 | 4 | 4 | 4 | 4 | 4 |
| 50 | LH1 | 4 | 4 | 4 | 4 | 4 | 4 |
| 51 | LH1 | 4 | 4 | 3 | 4 | 3 | 4 |
| 52 | LH1 | 4 | 4 | 4 | 4 | 4 | 4 |
| 53 | LH1 | 4 | 4 | 3 | 4 | 3 | 4 |
| 54 | LH1 | 4 | 4 | 4 | 4 | 4 | 4 |
| 55 | LH1 | 4 | 4 | 4 | 4 | 4 | 4 |
| 56 | LH1 | 3 | 4 | 4 | 3 | 4 | 4 |
| 57 | LH1 | 4 | 4 | 4 | 4 | 4 | 4 |
| 58 | LH1 | 4 | 4 | 4 | 4 | 4 | 3 |
| 59 | LH1 | 4 | 4 | 3 | 4 | 4 | 4 |
| 60 | LH1 | 3 | 4 | 4 | 3 | 4 | 4 |
| 61 | LH1 | 4 | 4 | 4 | 4 | 4 | 4 |
| 62 | LH1 | 4 | 4 | 4 | 4 | 4 | 4 |
| 63 | LH1 | 4 | 4 | 4 | 3 | 3 | 4 |
| 64 | LH1 | 4 | 4 | 4 | 4 | 4 | 4 |
| 65 | LH1 | 4 | 4 | 4 | 4 | 4 | 3 |
| 66 | LH1 | 4 | 4 | 4 | 4 | 4 | 4 |
| 67 | LH1 | 3 | 4 | 4 | 4 | 4 | 4 |
| 68 | LH1 | 4 | 4 | 4 | 4 | 4 | 4 |
| 69 | LH1 | 4 | 4 | 4 | 4 | 4 | 4 |
| 70 | LH1 | 4 | 4 | 4 | 4 | 4 | 4 |
| 71 | LH1 | 4 | 4 | 4 | 4 | 4 | 4 |
| 72 | LH1 | 4 | 4 | 4 | 4 | 4 | 4 |
| 73 | LH1 | 4 | 3 | 4 | 4 | 4 | 4 |
| 74 | LH1 | 4 | 4 | 4 | 4 | 4 | 4 |
| 75 | LH1 | 3 | 4 | 4 | 4 | 4 | 4 |
| 76 | LH1 | 4 | 4 | 3 | 4 | 4 | 4 |
| 77 | LH1 | 4 | 4 | 4 | 4 | 4 | 4 |
| 78 | LH1 | 4 | 4 | 4 | 4 | 4 | 4 |
| 79 | LH1 | 4 | 4 | 4 | 4 | 4 | 4 |
| 80 | LH1 | 4 | 4 | 4 | 4 | 4 | 4 |
| 81 | LH1 | 3 | 2 | 4 | 3 | 4 | 4 |
| 82 | LH1 | 4 | 4 | 4 | 4 | 4 | 4 |
| 83 | LH1 | 4 | 4 | 4 | 4 | 4 | 4 |
| 84 | LH1 | 4 | 4 | 4 | 4 | 4 | 4 |
| 85 | LH1 | 4 | 4 | 4 | 4 | 4 | 3 |
| 86 | LH1 | 4 | 4 | 4 | 4 | 4 | 4 |
| 87 | LH1 | 3 | 3 | 4 | 2 | 4 | 4 |
| 1 | LH2 | 0 | 0 | 0 | 0 | 0 | 0 |
| 2 | LH2 | 0 | 0 | 0 | 0 | 0 | 0 |
| 3 | LH2 | 0 | 0 | 0 | 0 | 0 | 0 |
| 4 | LH2 | 0 | 0 | 0 | 0 | 0 | 0 |
| 5 | LH2 | 0 | 0 | 0 | 0 | 0 | 0 |
| 6 | LH2 | 0 | 0 | 0 | 0 | 0 | 0 |
| 7 | LH2 | 0 | 0 | 0 | 0 | 0 | 0 |
| 8 | LH2 | 0 | 0 | 0 | 0 | 0 | 0 |
| 9 | LH2 | 0 | 0 | 0 | 0 | 0 | 0 |
| 10 | LH2 | 0 | 0 | 0 | 0 | 0 | 0 |
| 11 | LH2 | 0 | 0 | 0 | 0 | 0 | 0 |
| 12 | LH2 | 0 | 0 | 0 | 0 | 0 | 0 |
| 13 | LH2 | 0 | 0 | 0 | 0 | 0 | 0 |
| 14 | LH2 | 0 | 0 | 0 | 0 | 0 | 0 |
| 15 | LH2 | 0 | 0 | 0 | 0 | 0 | 0 |
| 16 | LH2 | 0 | 0 | 0 | 0 | 0 | 0 |
| 17 | LH2 | 0 | 0 | 0 | 0 | 0 | 0 |
| 18 | LH2 | 0 | 0 | 0 | 0 | 0 | 0 |
| 19 | LH2 | 0 | 0 | 0 | 0 | 0 | 0 |
| 20 | LH2 | 0 | 0 | 0 | 0 | 0 | 0 |
| 21 | LH2 | 0 | 0 | 0 | 0 | 0 | 0 |
| 22 | LH2 | 0 | 0 | 0 | 0 | 0 | 0 |
| 23 | LH2 | 0 | 0 | 0 | 0 | 0 | 0 |
| 24 | LH2 | 0 | 0 | 0 | 0 | 0 | 0 |
| 25 | LH2 | 0 | 0 | 0 | 0 | 0 | 0 |
| 26 | LH2 | 0 | 0 | 0 | 0 | 0 | 0 |
| 27 | LH2 | 0 | 0 | 0 | 0 | 0 | 0 |
| 28 | LH2 | 0 | 0 | 0 | 0 | 0 | 0 |
| 29 | LH2 | 0 | 0 | 0 | 0 | 0 | 0 |
| 30 | LH2 | 0 | 0 | 0 | 0 | 0 | 0 |
| 31 | LH2 | 0 | 0 | 0 | 0 | 0 | 0 |
| 32 | LH2 | 0 | 0 | 0 | 0 | 0 | 0 |
| 33 | LH2 | 0 | 0 | 0 | 0 | 0 | 0 |
| 34 | LH2 | 0 | 0 | 0 | 0 | 0 | 0 |
| 35 | LH2 | 0 | 0 | 0 | 0 | 0 | 0 |
| 36 | LH2 | 0 | 0 | 0 | 0 | 0 | 0 |
| 37 | LH2 | 0 | 0 | 0 | 0 | 0 | 0 |
| 38 | LH2 | 0 | 0 | 0 | 0 | 0 | 0 |
| 39 | LH2 | 0 | 0 | 0 | 0 | 0 | 0 |
| 40 | LH2 | 0 | 0 | 0 | 0 | 0 | 0 |
| 41 | LH2 | 0 | 0 | 0 | 0 | 0 | 0 |
| 42 | LH2 | 0 | 0 | 0 | 0 | 0 | 0 |
| 43 | LH2 | 0 | 0 | 0 | 0 | 0 | 0 |
| 44 | LH2 | 0 | 0 | 0 | 0 | 0 | 0 |
| 45 | LH2 | 0 | 0 | 0 | 0 | 0 | 0 |
| 46 | LH2 | 0 | 0 | 0 | 0 | 0 | 0 |
| 47 | LH2 | 0 | 0 | 0 | 0 | 0 | 0 |
| 48 | LH2 | 0 | 0 | 0 | 0 | 0 | 0 |
| 49 | LH2 | 0 | 0 | 0 | 0 | 0 | 0 |
| 50 | LH2 | 0 | 0 | 0 | 0 | 0 | 0 |
| 51 | LH2 | 0 | 0 | 0 | 0 | 0 | 0 |
| 52 | LH2 | 0 | 0 | 0 | 0 | 0 | 0 |
| 53 | LH2 | 0 | 0 | 0 | 0 | 0 | 0 |
| 54 | LH2 | 0 | 0 | 0 | 0 | 0 | 0 |
| 55 | LH2 | 0 | 0 | 0 | 0 | 0 | 0 |
| 56 | LH2 | 0 | 0 | 0 | 0 | 0 | 0 |
| 57 | LH2 | 0 | 0 | 0 | 0 | 0 | 0 |
| 58 | LH2 | 0 | 0 | 0 | 0 | 0 | 0 |
| 59 | LH2 | 0 | 0 | 0 | 0 | 0 | 0 |
| 60 | LH2 | 0 | 0 | 0 | 0 | 0 | 0 |
| 61 | LH2 | 0 | 0 | 0 | 0 | 0 | 0 |
| 62 | LH2 | 0 | 0 | 0 | 0 | 0 | 0 |
| 63 | LH2 | 0 | 0 | 0 | 0 | 0 | 0 |
| 64 | LH2 | 0 | 0 | 0 | 0 | 0 | 0 |
| 65 | LH2 | 0 | 0 | 0 | 0 | 0 | 0 |
| 66 | LH2 | 0 | 0 | 0 | 0 | 0 | 0 |
| 67 | LH2 | 0 | 0 | 0 | 0 | 0 | 0 |
| 68 | LH2 | 0 | 0 | 0 | 0 | 0 | 0 |
| 69 | LH2 | 0 | 0 | 0 | 0 | 0 | 0 |
| 70 | LH2 | 0 | 0 | 0 | 0 | 0 | 0 |
| 71 | LH2 | 0 | 0 | 0 | 0 | 0 | 0 |
| 72 | LH2 | 0 | 0 | 0 | 0 | 0 | 0 |
| 73 | LH2 | 0 | 0 | 0 | 0 | 0 | 0 |
| 74 | LH2 | 0 | 0 | 0 | 0 | 0 | 0 |
| 75 | LH2 | 0 | 0 | 0 | 0 | 0 | 0 |
| 76 | LH2 | 0 | 0 | 0 | 0 | 0 | 0 |
| 77 | LH2 | 0 | 0 | 0 | 0 | 0 | 0 |
| 78 | LH2 | 0 | 0 | 0 | 0 | 0 | 0 |
| 79 | LH2 | 0 | 0 | 0 | 0 | 0 | 0 |
| 80 | LH2 | 0 | 0 | 0 | 0 | 0 | 0 |
| 81 | LH2 | 0 | 0 | 0 | 0 | 0 | 0 |
| 82 | LH2 | 0 | 0 | 0 | 0 | 0 | 0 |
| 83 | LH2 | 0 | 0 | 0 | 0 | 0 | 0 |
| 84 | LH2 | 0 | 0 | 0 | 0 | 0 | 0 |
| 85 | LH2 | 0 | 0 | 0 | 0 | 0 | 0 |
| 86 | LH2 | 0 | 0 | 0 | 0 | 0 | 0 |
| 87 | LH2 | 0 | 0 | 0 | 0 | 0 | 0 |
| 1 | LH3 | 4 | 4 | 4 | 3 | 4 | 4 |
| 2 | LH3 | 4 | 4 | 4 | 4 | 4 | 4 |
| 3 | LH3 | 4 | 4 | 4 | 4 | 4 | 4 |
| 4 | LH3 | 4 | 3 | 4 | 4 | 4 | 4 |
| 5 | LH3 | 4 | 4 | 4 | 4 | 4 | 4 |
| 6 | LH3 | 4 | 4 | 4 | 4 | 4 | 4 |
| 7 | LH3 | 4 | 4 | 4 | 4 | 4 | 4 |
| 8 | LH3 | 4 | 4 | 3 | 4 | 4 | 4 |
| 9 | LH3 | 4 | 4 | 4 | 4 | 4 | 4 |
| 10 | LH3 | 4 | 4 | 4 | 4 | 4 | 4 |
| 11 | LH3 | 4 | 4 | 4 | 4 | 4 | 4 |
| 12 | LH3 | 3 | 4 | 4 | 4 | 4 | 4 |
| 13 | LH3 | 4 | 4 | 4 | 3 | 4 | 4 |
| 14 | LH3 | 4 | 4 | 4 | 4 | 4 | 4 |
| 15 | LH3 | 4 | 4 | 4 | 4 | 4 | 4 |
| 16 | LH3 | 4 | 4 | 4 | 4 | 4 | 4 |
| 17 | LH3 | 4 | 4 | 3 | 4 | 4 | 4 |
| 18 | LH3 | 4 | 4 | 4 | 4 | 4 | 4 |
| 19 | LH3 | 4 | 4 | 4 | 4 | 4 | 4 |
| 20 | LH3 | 4 | 4 | 4 | 4 | 3 | 4 |
| 21 | LH3 | 4 | 4 | 4 | 4 | 4 | 4 |
| 22 | LH3 | 4 | 4 | 4 | 4 | 4 | 4 |
| 23 | LH3 | 4 | 4 | 4 | 4 | 4 | 4 |
| 24 | LH3 | 3 | 4 | 4 | 4 | 4 | 4 |
| 25 | LH3 | 4 | 4 | 4 | 4 | 4 | 4 |
| 26 | LH3 | 4 | 4 | 4 | 4 | 4 | 4 |
| 27 | LH3 | 4 | 3 | 4 | 4 | 4 | 4 |
| 28 | LH3 | 4 | 4 | 4 | 4 | 4 | 4 |
| 29 | LH3 | 4 | 4 | 4 | 4 | 4 | 4 |
| 30 | LH3 | 4 | 4 | 4 | 4 | 4 | 4 |
| 31 | LH3 | 4 | 4 | 3 | 4 | 4 | 4 |
| 32 | LH3 | 4 | 4 | 4 | 4 | 4 | 4 |
| 33 | LH3 | 4 | 4 | 4 | 4 | 4 | 4 |
| 34 | LH3 | 4 | 4 | 4 | 4 | 4 | 4 |
| 35 | LH3 | 4 | 4 | 3 | 4 | 4 | 4 |
| 36 | LH3 | 4 | 4 | 4 | 4 | 4 | 4 |
| 37 | LH3 | 3 | 4 | 4 | 4 | 4 | 4 |
| 38 | LH3 | 4 | 4 | 4 | 4 | 4 | 4 |
| 39 | LH3 | 4 | 4 | 4 | 4 | 4 | 3 |
| 40 | LH3 | 4 | 4 | 4 | 4 | 4 | 4 |
| 41 | LH3 | 4 | 4 | 4 | 4 | 4 | 4 |
| 42 | LH3 | 4 | 4 | 4 | 4 | 4 | 4 |
| 43 | LH3 | 4 | 4 | 4 | 4 | 4 | 4 |
| 44 | LH3 | 3 | 4 | 4 | 4 | 4 | 4 |
| 45 | LH3 | 3 | 4 | 4 | 4 | 4 | 4 |
| 46 | LH3 | 4 | 4 | 4 | 4 | 4 | 4 |
| 47 | LH3 | 4 | 4 | 4 | 4 | 4 | 4 |
| 48 | LH3 | 4 | 4 | 4 | 4 | 4 | 4 |
| 49 | LH3 | 4 | 4 | 4 | 4 | 4 | 4 |
| 50 | LH3 | 4 | 4 | 4 | 4 | 4 | 4 |
| 51 | LH3 | 4 | 4 | 4 | 4 | 4 | 4 |
| 52 | LH3 | 4 | 4 | 4 | 4 | 4 | 4 |
| 53 | LH3 | 4 | 4 | 3 | 4 | 3 | 4 |
| 54 | LH3 | 4 | 4 | 4 | 4 | 4 | 4 |
| 55 | LH3 | 4 | 4 | 4 | 4 | 4 | 4 |
| 56 | LH3 | 4 | 4 | 3 | 4 | 4 | 3 |
| 57 | LH3 | 4 | 4 | 4 | 4 | 4 | 4 |
| 58 | LH3 | 4 | 4 | 4 | 4 | 4 | 4 |
| 59 | LH3 | 4 | 4 | 4 | 4 | 4 | 3 |
| 60 | LH3 | 4 | 4 | 4 | 4 | 4 | 4 |
| 61 | LH3 | 4 | 2 | 4 | 4 | 4 | 4 |
| 62 | LH3 | 4 | 4 | 4 | 4 | 4 | 4 |
| 63 | LH3 | 4 | 4 | 4 | 4 | 4 | 4 |
| 64 | LH3 | 4 | 4 | 4 | 4 | 4 | 4 |
| 65 | LH3 | 4 | 4 | 4 | 3 | 4 | 4 |
| 66 | LH3 | 4 | 4 | 4 | 4 | 4 | 4 |
| 67 | LH3 | 4 | 4 | 4 | 4 | 4 | 4 |
| 68 | LH3 | 4 | 4 | 4 | 4 | 4 | 4 |
| 69 | LH3 | 4 | 4 | 4 | 4 | 4 | 4 |
| 70 | LH3 | 4 | 3 | 4 | 4 | 4 | 4 |
| 71 | LH3 | 4 | 4 | 4 | 4 | 4 | 4 |
| 72 | LH3 | 4 | 4 | 4 | 4 | 4 | 4 |
| 73 | LH3 | 4 | 4 | 4 | 4 | 4 | 4 |
| 74 | LH3 | 4 | 4 | 4 | 4 | 4 | 4 |
| 75 | LH3 | 4 | 4 | 4 | 4 | 4 | 4 |
| 76 | LH3 | 4 | 4 | 3 | 4 | 4 | 4 |
| 77 | LH3 | 4 | 4 | 4 | 4 | 4 | 4 |
| 78 | LH3 | 4 | 4 | 4 | 4 | 4 | 4 |
| 79 | LH3 | 4 | 4 | 4 | 4 | 4 | 3 |
| 80 | LH3 | 4 | 4 | 4 | 4 | 4 | 4 |
| 81 | LH3 | 4 | 4 | 4 | 3 | 4 | 4 |
| 82 | LH3 | 4 | 4 | 4 | 4 | 4 | 4 |
| 83 | LH3 | 4 | 4 | 4 | 4 | 4 | 4 |
| 84 | LH3 | 4 | 4 | 4 | 4 | 4 | 3 |
| 85 | LH3 | 4 | 4 | 4 | 4 | 4 | 4 |
| 86 | LH3 | 4 | 4 | 4 | 4 | 4 | 4 |
| 87 | LH3 | 4 | 4 | 4 | 4 | 4 | 4 |
| 1 | LH4 | 4 | 4 | 4 | 4 | 4 | 4 |
| 2 | LH4 | 4 | 4 | 4 | 4 | 4 | 4 |
| 3 | LH4 | 4 | 4 | 4 | 4 | 4 | 3 |
| 4 | LH4 | 4 | 4 | 4 | 4 | 4 | 4 |
| 5 | LH4 | 4 | 4 | 4 | 3 | 4 | 4 |
| 6 | LH4 | 4 | 4 | 4 | 4 | 4 | 4 |
| 7 | LH4 | 4 | 3 | 4 | 4 | 4 | 4 |
| 8 | LH4 | 4 | 4 | 4 | 4 | 4 | 4 |
| 9 | LH4 | 4 | 4 | 4 | 4 | 4 | 4 |
| 10 | LH4 | 4 | 4 | 4 | 4 | 4 | 4 |
| 11 | LH4 | 4 | 4 | 4 | 4 | 4 | 4 |
| 12 | LH4 | 4 | 4 | 3 | 4 | 4 | 4 |
| 13 | LH4 | 2 | 2 | 4 | 4 | 4 | 4 |
| 14 | LH4 | 4 | 4 | 4 | 4 | 4 | 4 |
| 15 | LH4 | 4 | 4 | 4 | 4 | 4 | 4 |
| 16 | LH4 | 4 | 4 | 4 | 4 | 4 | 4 |
| 17 | LH4 | 4 | 4 | 4 | 4 | 4 | 4 |
| 18 | LH4 | 4 | 4 | 4 | 4 | 4 | 4 |
| 19 | LH4 | 4 | 4 | 4 | 4 | 4 | 4 |
| 20 | LH4 | 4 | 3 | 4 | 3 | 4 | 4 |
| 21 | LH4 | 4 | 4 | 3 | 4 | 4 | 4 |
| 22 | LH4 | 4 | 4 | 4 | 4 | 4 | 4 |
| 23 | LH4 | 4 | 4 | 4 | 4 | 4 | 4 |
| 24 | LH4 | 4 | 4 | 4 | 4 | 4 | 4 |
| 25 | LH4 | 4 | 3 | 4 | 4 | 4 | 3 |
| 26 | LH4 | 4 | 4 | 4 | 4 | 4 | 4 |
| 27 | LH4 | 4 | 4 | 4 | 4 | 4 | 4 |
| 28 | LH4 | 4 | 4 | 4 | 4 | 4 | 4 |
| 29 | LH4 | 4 | 4 | 4 | 4 | 4 | 4 |
| 30 | LH4 | 4 | 3 | 4 | 4 | 4 | 4 |
| 31 | LH4 | 4 | 4 | 4 | 4 | 4 | 4 |
| 32 | LH4 | 4 | 4 | 4 | 4 | 4 | 4 |
| 33 | LH4 | 4 | 4 | 4 | 4 | 4 | 4 |
| 34 | LH4 | 4 | 4 | 4 | 4 | 4 | 4 |
| 35 | LH4 | 4 | 4 | 4 | 4 | 4 | 4 |
| 36 | LH4 | 2 | 4 | 4 | 4 | 4 | 4 |
| 37 | LH4 | 4 | 4 | 4 | 4 | 4 | 4 |
| 38 | LH4 | 4 | 4 | 4 | 4 | 4 | 4 |
| 39 | LH4 | 4 | 4 | 4 | 4 | 4 | 4 |
| 40 | LH4 | 4 | 4 | 4 | 4 | 4 | 4 |
| 41 | LH4 | 4 | 4 | 4 | 4 | 4 | 4 |
| 42 | LH4 | 4 | 4 | 4 | 4 | 4 | 4 |
| 43 | LH4 | 4 | 4 | 4 | 4 | 4 | 4 |
| 44 | LH4 | 4 | 4 | 4 | 4 | 4 | 4 |
| 45 | LH4 | 4 | 3 | 4 | 4 | 4 | 4 |
| 46 | LH4 | 4 | 4 | 4 | 4 | 4 | 4 |
| 47 | LH4 | 4 | 4 | 4 | 4 | 4 | 4 |
| 48 | LH4 | 4 | 4 | 4 | 4 | 4 | 4 |
| 49 | LH4 | 4 | 3 | 4 | 4 | 4 | 4 |
| 50 | LH4 | 4 | 4 | 4 | 4 | 4 | 4 |
| 51 | LH4 | 4 | 4 | 4 | 4 | 4 | 4 |
| 52 | LH4 | 4 | 4 | 4 | 4 | 4 | 4 |
| 53 | LH4 | 4 | 4 | 4 | 4 | 4 | 4 |
| 54 | LH4 | 4 | 4 | 4 | 4 | 4 | 4 |
| 55 | LH4 | 4 | 4 | 3 | 4 | 4 | 4 |
| 56 | LH4 | 4 | 4 | 4 | 4 | 4 | 4 |
| 57 | LH4 | 3 | 4 | 4 | 4 | 4 | 4 |
| 58 | LH4 | 4 | 4 | 4 | 4 | 4 | 4 |
| 59 | LH4 | 4 | 4 | 4 | 4 | 4 | 4 |
| 60 | LH4 | 4 | 3 | 4 | 4 | 4 | 4 |
| 61 | LH4 | 4 | 4 | 2 | 2 | 4 | 4 |
| 62 | LH4 | 4 | 4 | 4 | 4 | 4 | 4 |
| 63 | LH4 | 4 | 4 | 4 | 4 | 4 | 4 |
| 64 | LH4 | 4 | 4 | 4 | 4 | 4 | 3 |
| 65 | LH4 | 4 | 4 | 4 | 4 | 4 | 4 |
| 66 | LH4 | 4 | 4 | 4 | 4 | 4 | 4 |
| 67 | LH4 | 4 | 4 | 4 | 4 | 4 | 4 |
| 68 | LH4 | 4 | 4 | 4 | 4 | 4 | 4 |
| 69 | LH4 | 4 | 4 | 4 | 4 | 4 | 4 |
| 70 | LH4 | 3 | 4 | 4 | 4 | 4 | 4 |
| 71 | LH4 | 4 | 4 | 4 | 4 | 4 | 4 |
| 72 | LH4 | 4 | 4 | 4 | 4 | 4 | 4 |
| 73 | LH4 | 4 | 4 | 4 | 4 | 4 | 4 |
| 74 | LH4 | 4 | 4 | 4 | 4 | 4 | 4 |
| 75 | LH4 | 4 | 4 | 4 | 4 | 4 | 4 |
| 76 | LH4 | 4 | 4 | 4 | 4 | 4 | 4 |
| 77 | LH4 | 4 | 4 | 4 | 4 | 4 | 4 |
| 78 | LH4 | 4 | 4 | 4 | 4 | 4 | 4 |
| 79 | LH4 | 4 | 4 | 4 | 3 | 4 | 4 |
| 80 | LH4 | 4 | 4 | 4 | 4 | 4 | 4 |
| 81 | LH4 | 3 | 4 | 3 | 4 | 4 | 4 |
| 82 | LH4 | 4 | 4 | 4 | 4 | 4 | 4 |
| 83 | LH4 | 4 | 4 | 4 | 4 | 4 | 4 |
| 84 | LH4 | 4 | 4 | 4 | 4 | 4 | 3 |
| 85 | LH4 | 4 | 4 | 4 | 4 | 4 | 4 |
| 86 | LH4 | 4 | 4 | 4 | 4 | 4 | 4 |
| 87 | LH4 | 4 | 4 | 4 | 4 | 4 | 4 |
